# Inhibition of neutrophil respiratory burst and degranulation responses by CVT-E002, the main active ingredient in COLD-FX

**DOI:** 10.1186/1710-1492-7-S2-A31

**Published:** 2011-11-14

**Authors:** Renjith Pillai, Paige Lacy

**Affiliations:** 1 Pulmonary Research Group, Department of Medicine, University of Alberta, Edmonton, AB, Canada T6G 2S2

## Background

Human peripheral blood neutrophils contribute to the first line of defence in the immune system and are critical for maintaining health and immunity against opportunistic infections. Neutrophils and their granule-derived mediators are frequently found elevated in patient samples in viral infections, asthma exacerbations, and other respiratory ailments. COLD-FX has been shown to reduce the symptoms and severity of respiratory tract viral infections. Our hypothesis is that COLD-FX modulates neutrophil activity. To determine the effects of COLD-FX on neutrophils, peripheral blood neutrophils (>97% purity) were isolated from healthy human volunteers. 

## Methods

Neutrophils were preincubated with varying doses of CVT-E002 (0.01-1 mg/ml), the active ingredient of COLD-FX, for 30, 60, and 120 min. Extracellular ROS production was measured by cytochrome c reduction from neutrophils stimulated with 50 ng/ml phorbol myristate acetate for up to 60 min. Degranulation was measured by the presence of extracellular myeloperoxidase, a marker of the azurophilic granules, in neutrophils stimulated with cytochalasin B and f-Met-Leu-Phe for 15 min.

## Results

CVT-E002 (1 mg/ml) had no significant effect on viability at up to 120 min of incubation. At 60 min of incubation with CVT-E002, neutrophils showed a 30% reduction in ROS generation (p < 0.001) which was maintained for up to 120 min. Preliminary experiments also showed that incubation of neutrophils with CVT-E002 for 30 min inhibited myeloperoxidase release.

## Conclusions

These novel findings demonstrate that COLD-FX significantly reduces activation of neutrophils. The implications of this study are that COLD-FX may reduce oxidative stress and tissue-damage triggered by neutrophilic inflammation and activation.

Funding source: Afexa Life Sciences, Inc.

